# THE CUTTING EDGE OF GERONTOLOGICAL SCHOLARSHIP: THE GERONTOLOGIST’S EDITOR’S CHOICE SYMPOSIUM

**DOI:** 10.1093/geroni/igae098.1550

**Published:** 2024-12-31

**Authors:** Joseph Gaugler, Alexandrea Allemann

**Affiliations:** Chair; Co-Chair

## Abstract

The mission of The Gerontologist is to publish and disseminate “applied, multidisciplinary research and analysis on social issues related to human aging. It informs the broad community of disciplines and professions involved in understanding the aging process and providing care to older people.” In addition, the journal encourages “cutting-edge, conceptually-oriented work that addresses inequities in health, mental health, social status, and justice in late life as well as intersections among these areas with applicability to real-world-settings.” This symposium will highlight some of the recent and best examples of gerontological scholarship in The Gerontologist by highlighting select Editor’s Choice articles. Andrea Gilmore-Bykovskyi, Ph.D., RN, and colleagues consider the perspectives of family caregivers and hospice clinicians related to episodes of paradoxical lucidity among people living with dementia and how observational approaches may be leveraged to understand this fascinating phenomenon further. Kelly O’Malley, Ph.D., and her co-authors offer a timely and important framework to advance and implement trauma-informed care in long-term care contexts. Ittay Mannheim, Ph.D., and colleagues conducted a fascinating scoping review using critical discourse analysis to determine how and the extent to which ageism is reflected in the design process of digital technologies. Together, these Editor’s Choice selections from The Gerontologist “push the envelope” of the science and scholarship of gerontology.

